# Z-ajoene from Crushed Garlic Alleviates Cancer-Induced Skeletal Muscle Atrophy

**DOI:** 10.3390/nu11112724

**Published:** 2019-11-10

**Authors:** Hyejin Lee, Ji-Won Heo, A-Reum Kim, Minson Kweon, Sorim Nam, Jong-Seok Lim, Mi-Kyung Sung, Sung-Eun Kim, Jae-Ha Ryu

**Affiliations:** 1 Research Institute of Pharmaceutical Sciences, Sookmyung Women’s University, Yongsan-Gu, Seoul 04310, Korea; u9698115@naver.com (H.L.); minson-_-@nate.com (M.K.); 2 Department of Food and Nutrition, Sookmyung Women’s University, Yongsan-Gu, Seoul 04310, Korea; hjiwon1021@naver.com (J.-W.H.); aarmy92@naver.com (A.-R.K.); mksung@sookmyung.ac.kr (M.-K.S.); 3 Division of Biological Sciences and Cellular Heterogeneity Research Center, Sookmyung Women’s University, Yongsan-Gu, Seoul 04310, Korea; sorim.nam@gmail.com (S.N.); jslim@sookmyung.ac.kr (J.-S.L.)

**Keywords:** *Allium sativum*, Z-ajoene, cancer, skeletal muscle atrophy, C2C12 cells

## Abstract

Skeletal muscle atrophy is one of the major symptoms of cancer cachexia. Garlic (*Allium sativum*), one of the world’s most commonly used and versatile herbs, has been employed for the prevention and treatment of diverse diseases for centuries. In the present study, we found that ajoene, a sulfur compound found in crushed garlic, exhibits protective effects against muscle atrophy. Using CT26 tumor-bearing BALB/c mice, we demonstrate in vivo that ajoene extract alleviated muscle degradation by decreasing not only myokines secretion but also janus kinase/signal transducer and activator of transcription 3 (JAK/STAT3) and SMADs/forkhead box (FoxO) signaling pathways, thereby suppressing muscle-specific E3 ligases. In mouse skeletal myoblasts, Z-ajoene enhanced myogenesis as evidenced by increased expression of myogenic markers via p38 mitogen-activated protein kinase (MAPK) activation. In mature myotubes, Z-ajoene protected against muscle protein degradation induced by conditioned media from CT26 colon carcinoma cells, by suppressing expression of muscle specific E3 ligases and nuclear transcription factor kappa B (NF-κB) phosphorylation which contribute to muscle atrophy. Moreover, Z-ajoene treatment improved myofiber formation via stimulation of muscle protein synthesis. These findings suggest that ajoene extract and Z-ajoene can attenuate skeletal muscle atrophy induced by cancer cachexia through suppressing inflammatory responses and the muscle wasting as well as by promoting muscle protein synthesis.

## 1. Introduction

Cancer is one of the leading causes of morbidity and mortality. More than 18 million new cases were estimated to have been diagnosed in 2018 [1]. Around 50–80% of cancer patients, particularly those with gastrointestinal cancer including colon cancer, suffer from cachexia, which is a complex metabolic syndrome. Cachexia is characterized by weight loss mainly as a result of progressive loss of skeletal muscle, leading to inevitable functional impairment [2,3,4]. Cancer cachexia is also associated with poor responses to chemotherapy and quality of life, thereby contributing to shortened survival times [3,4]. Since at least 20–40% of cancer deaths are attributable to cachexia [3,5], there is an urgent need to find effective strategies for preventing and treating these symptoms in cancer patients.

Skeletal muscle atrophy is considered to be the representative manifestation of cancer cachexia and results from an imbalance of protein synthesis and degradation [4,6]. Specifically in cachexia, there appears to be activation of proteolytic pathways, which are primarily regulated by the ubiquitin-proteasome pathway and the lysosomal autophagy system [7,8,9]. In muscle, the ubiquitin-proteasome pathway is mediated by E3 ligases, particularly the muscle atrophy F-box protein 1 (MAFbx) and the muscle RING finger containing protein 1 (MuRF1) [8,9]. Pro-inflammatory cytokines such as interleukin-6 (IL-6) contribute to the activation of nuclear transcription factor kappa B (NF-κB) and janus kinase/signal transducer and activator of transcription 3 (JAK/STAT3) signaling, followed by the induction of muscle-specific E3 ligases [4,10]. In addition, transforming growth factor β (TGF-β) family members such as myostatin are known to stimulate muscle atrophy through the SMADs/forkhead box (FoxO) network. SMAD2/3 was reported to be crucial for the activation of FoxO1, leading to MAFbx transcription [11]. Accordingly, protein degradation, inflammatory cytokines, and myostatin were suggested as therapeutic targets for muscle atrophy [12,13,14], but to date, no therapeutic agents has been approved for clinical use. 

Despite ongoing efforts to develop new treatments for muscle atrophy, only megestrol acetate has been approved by the US Food and Drug Administration as an anti-myopathy drug. Given that cancer cachexia is a multifactorial condition, multimodal therapeutic approaches, such as nutritional supplements that incorporate nutraceuticals, can be a strategy for the prevention and treatment of cachexia [3,4,15]. Growing evidence, including our previous reports, has highlighted the beneficial counteracting effects of several natural products on muscle atrophy [16]. 

Garlic is a widely used flavoring agent in food and has been a well utilized remedy for numerous ailments over thousands of years. It is also considered to be one of the most powerful chemopreventive and anti-cancer foods [17]. Recently, the anti-fatigue potential of processed garlic against exercise-induced fatigue was reported in mice suggesting regenerative properties for muscle [18]. From many studies for the medicinal applications of garlic [19,20], more than 33 sulfur compounds have been identified and of these, allicin is one of the most well-known. Another attractive sulfur compound, ajoene, is only found in the crushed garlic bulb. Ajoene is transformed from allicin which is derived from alliin by 2-allinase-induced bond cleavage and is chemically more stable than allicin. Broad biological activities have been ascribed to ajoene, including antithrombotic, antimicrobial, and anticancer activities and these have attracted attention in efforts to develop herbal supplements or medicines [21]. We previously reported that Z-ajoene has antioxidant activity through Nrf2-mediated glutamate-cysteine ligase induction [22,23]. We also reported the anti-inflammatory activity of Z-ajoene via suppressing NF-kB pathway [24]. We assume that these bio-activities of Z-ajoene might contribute to the muscle protection under the damaged condition [25].

In the present study, we investigated the effects of Z-ajoene from crushed garlic (*Allium sativum*) on skeletal muscle atrophy and described underlying mechanisms in a mouse model of colon cancer cachexia and a murine myoblast cell line. 

## 2. Materials and Methods

### 2.1. Preparation of Ajoene Extract and Purification of Z-Ajoene from Garlic

Garlic (*Allium sativum* L.) (2 kg) was purchased from a Korean market and samples have been deposited as voucher specimen (No. SPH-1803) in herbarium of Sookmyung Women’s University. Garlic bulb was crushed and incubated at room temperature for 1 h and extracted with ethyl acetate at 60 °C for 8 h. The ethyl acetate extract was evaporated in vacuo to make ajoene extract for animal study. The contents of Z-ajoene and E-ajoene were analyzed by high performance liquid chromatography (HPLC) as 11.1% (*w*/*w*) and 3.1% (*w*/*w*), respectively (Figure S1). Z-ajoene was isolated by repeated column chromatography and the purity was analyzed as higher than 98% by HPLC as reported previously [24]. 

### 2.2. Mouse Model of Cancer Cachexia

Six-week-old male BALB/c mice were obtained from Orient Bio (Sungnam, Republic of Korea). The animals were housed at 22 ± 1 °C under a 12 h light/dark cycle with ad libitum access to chow diet with water for 1 week. CT26 murine colon carcinoma cells were purchased from the Korean Cell Line Bank (KCLB; Seoul, Republic of Korea) and cultured in Dulbecco’s modified Eagle’s medium (DMEM) (WelGENE, Daegu, Republic of Korea) with 10% fetal bovine serum (Gibco BRL Life Technology, Grand Island, NY, USA). To induce cancer cachexia, BALB/c mice received subcutaneous injections of CT26 cells into the right flank of CT26 cells (5 × 10^5^ per mouse). After inoculation, the AIN-76A diet (Research Diets, Inc., New Brunswick, NJ, USA) was provided and body weight, food intake, and tumor volume were measured twice per week. The estimated tumor volume (V) was calculated based on the formula W^2^ × L × 0.5 (W, the largest tumor diameter in centimeters; L, the next largest tumor diameter in centimeters) as previously described [26]. When tumors reached 80–200 mm^3^ on day 15, mice were randomly assigned to three groups and received vehicle (saline; tumor control, TC), 5 mg/kg ajoene extract (A5), or 10 mg/kg ajoene extract (A10) intraperitoneally for a week (*n* = 10 per group). The experimental design is presented in Figure S2. At necropsy, tissues and serum were snap frozen in liquid nitrogen and stored at −80 °C until further experiments. This animal study was approved by the Institutional Animal Care and Use Committee of Sookmyung Women’s University (SMWU-IACUC-1702-049-03) and conducted in accordance with the Guide for the Care and Use of Laboratory Animals developed by the Institute of Laboratory Animal Resources of the National Research Council [27]. 

### 2.3. Assessment of Muscle Cross-Sectional Area

The quadriceps muscles were fixed in 4% paraformaldehyde and stained with hematoxylin and eosin (H&E) to measure the muscle fiber cross sectional area. After staining, 250 muscle fiber areas in a muscle section were averaged. Images were acquired by using Camera Nikon DS-Ri2 and analyzed using NIS-Elements BR 4.50.00 (Nikon, Tokyo, Japan). 

### 2.4. Flow Cytometry

Red blood cells (RBC) were removed from splenocytes using RBC lysing buffer (Sigma-Aldrich, St. Louis, MO, USA). The cells were incubated with antibodies for 30 min. The antibodies used for flow cytometry were as follows: CD45 (Tonbo Biosciences, San Diego, CA, USA), Gr-1, and CD11b (eBioscience, San Diego, CA, USA). Samples were acquired on a FACSCanto II (BD Biosciences, San Jose, CA, USA) using the Diva software. Data analysis was performed with the FlowJo software (Tree Star Inc., Ashland, OR, USA).

### 2.5. Cell Culture, Myoblast Differentiation and Collection of Conditioned Medium of CT26 Cancer Cells

C2C12 murine myoblast cells (American Type Culture Collection, Manassas, VA, USA) were maintained in growth medium (GM; DMEM containing 15% fetal bovine serum). When cells reached 95% confluence, GM was replaced with differentiation medium (DM, DMEM containing 2% horse serum) (differentiation day 0: D0). After 3 days (differentiation day 3: D3), cells were subjected to analytical experiments. 

To prepare the CT26 murine colon cancer cell-conditioned medium (CT26-CM), CT26 cells were seeded. After 24 h, cells were washed three times with phosphate-buffered saline (PBS) and replaced with serum-free DMEM to exclude serum inflammatory factors, followed by an additional 24 h incubation. The resulting CT26-CM was centrifuged, sterilized by filtering with a 0.22-μm syringe filter, and diluted into fresh DM, with a final concentration of 30% for cell treatment. 

### 2.6. Immunostaining of MHC

Myoblast or myotubes were fixed with 4% paraformaldehyde for 20 min and permeabilized with 0.1% Triton X-100 (Sigma-Aldrich, St. Louis, MO, USA) for 30 min. Then, the cells were incubated overnight at 4 °C with a primary antibody against myosin heavy chain (MHC) (MAB4470, R&D Systems, Minneapolis, MN, USA), followed by a goat anti-mouse antibody conjugated with Alexa Fluor 568 (LifeTechnologies, Carlsbad, CA, USA). In addition, cells were counterstained with DAPI (4′,6-diamidino-2-phenylindole) (Sigma-Aldrich, St. Louis, MO, USA) and the MHC immunofluorescence was detected under a fluorescence microscope (Olympus, Tokyo, Japan). Red fluorescence indicates MHC expression, and the multinucleated myotubes are observed with DAPI (blue-colored) counterstaining. 

### 2.7. RNA Extraction and Real-Time Quantitative Polymerase Chain Reaction Analysis (qRT-PCR)

Total RNA was extracted from mouse quadriceps muscle tissue and C2C12 cells using TRIzol reagent (Invitrogen™, Carlsbad, CA, USA). RNA purification and first-strand cDNA synthesis were performed following the manufacturer’s recommendation (Labopass™ cDNA synthesis kit, Cosmogenetech, Seoul, Republic of Korea). The RT-qPCR reaction was conducted with the SYBR^®^ Green PCR Master Mix and performed using an Applied Biosystems 7500 Fast Real-Time PCR System (Foster City, CA, USA). All mRNA levels were normalized to glyceraldehyde 3-phosphate dehydrogenase (GAPDH) mRNA levels. The primers used for the amplifications are presented in Table S1.

### 2.8. Western Blot Analysis

Following incubation, C2C12 cells were lysed and total proteins were subjected to Western blot analysis to analyze protein expression of myogenic markers and E3 ligases. The membrane was then incubated with antibodies specific to MHC (sc-376157, Santa Cruz, Dallas, TX, USA), myoD (sc-32758), myogenin (sc-12732), MAFbx (sc-166806), and MuRF1 (sc-398608). To investigate p38 MAPK activation by Z-ajoene, antibodies against phospho-p38 (9211, Cell Signaling Technology, Danvers, MA, USA) and p38 MAPK (9212) were used. Pan-cadherin (C3678, Sigma, St. Louis, MO, USA) was used as a loading control. 

### 2.9. Statistical Analysis

Differences were assessed using Student’s *t*-test or one-way analysis of variance (ANOVA) followed by the Duncan’s multiple range test with SAS version 9.4 (SAS Institute, Inc., Cary, NC, USA). All experiments were performed in triplicate at least three times. Differences with a *p* value of less than 0.05 were considered statistically significant.

## 3. Results

### 3.1. Ajoene Extract of Garlic Attenuates Cancer-Induced Muscle Atrophy in CT26 Tumor-Bearing Mice

**Figure 1:**
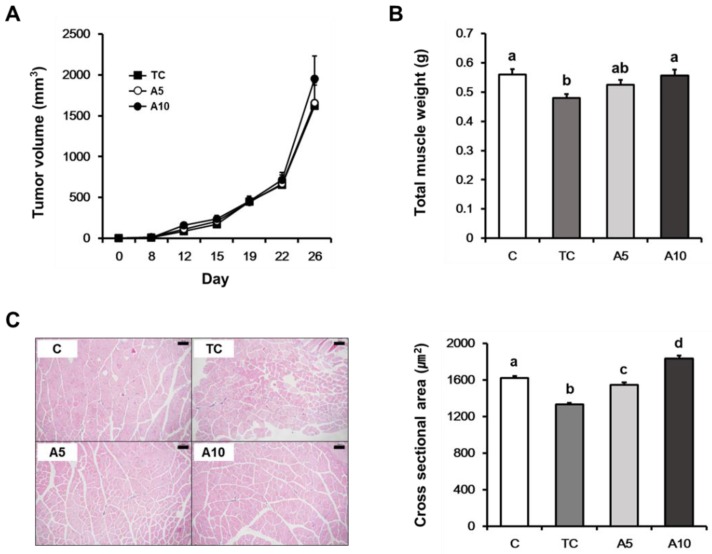
(**A**) Tumor volumes of CT26 tumor-bearing mice; (**B**) total muscle weight at the time of euthanasia; (**C**) representative hematoxylin and eosin (H&E) staining (magnification × 100) (left) and average value for cross sectional area (right) of the quadriceps muscle. Values are presented as the mean ± SEM. Statistical significance was evaluated by one-way ANOVA followed by the Duncan’s multiple range test. Means with different superscript letter are significantly different at *p* < 0.05 (**B**) and *p* < 0.0001 (**C**). C, control; TC, tumor control; A5, 5 mg/kg ajoene extract; A10, 10 mg/kg ajoene extract (*n* = 10 per group for **A** and **B**; n = 4 for **C**).

To investigate the effects of ajoene extract on cancer-induced muscle atrophy, we examined the in vivo efficacy of ajoene extract treatment in CT26 tumor-bearing mice. We did not observe significant differences in tumor growth among the mice treated with 0, 5, and 10 mg/kg ajoene extract (Figure 1A). Total muscle weight was significantly increased in the mice treated with 10 mg/kg of ajoene extract compared with the tumor control mice (*p* = 0.010) (Figure 1B; Table 1). Measurements of the cross-sectional area of the quadriceps muscle revealed that ajoene extract treatment (5 and 10 mg/kg) significantly increased the muscle fiber area compared with the tumor control group (*p* < 0.0001) (Figure 1C). In the cachexia groups, spleen and liver weights were increased compared with controls (*p* < 0.0001) and these did not differ among the three groups. The perirenal fat weight was reduced in the tumor control group, while it tended to increase in ajoene extract-treated groups (*p* = 0.020) (Table 1). Accordingly, these results indicate that ajoene extract treatment alleviates muscle atrophy at concentrations that do not exhibit anti-cancer effects in tumor-bearing mice. 

### 3.2. Ajoene Extract Suppresses Muscle Wasting by Reducing Myokines Secretion in CT26 Tumor-Bearing Mice

**Figure 2:**
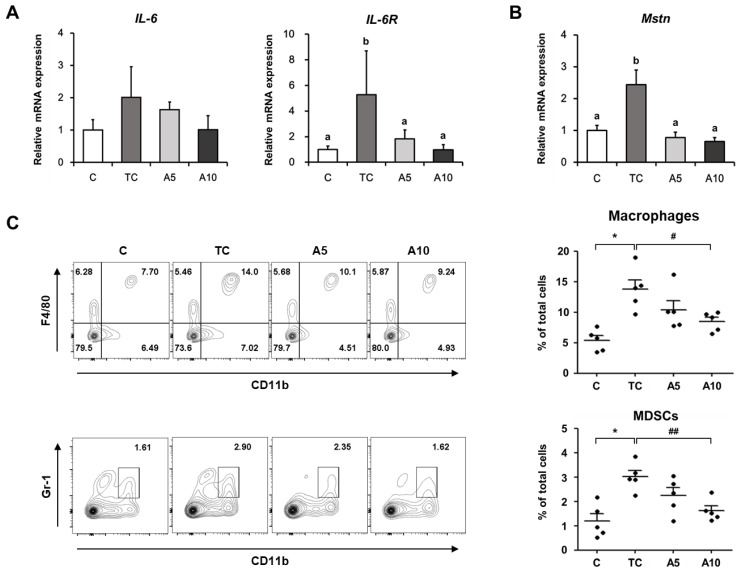
Effects of ajoene extract on myokines secretion in CT26 tumor-bearing mice. (**A**,**B**) mRNA expression of interleukin-6 (IL-6), interleukin-6 receptor (IL-6R), and myostatin in the quadriceps muscle; (**C**) representative contour plots showing the proportions of macrophages (top) and MDSCs (bottom) in the spleen. Values are presented as mean ± SEM. Statistical significance was evaluated by one-way ANOVA followed by the Duncan’s multiple range test or Student’s t-test. Means with different superscript letters are significantly different at *p* < 0.05 (**A**) and *p* < 0.001 (**B**). * *p* < 0.05 compared with the C group; # *p* < 0.05 and ## *p* < 0.01 compared with the TC group. MDSCs, myeloid-derived suppressor cells; C, control; TC, tumor control; A5, 5 mg/kg ajoene extract; A10, 10 mg/kg ajoene extract (*n* = 5 per group).

Recent studies have reported that myokines, which are secreted from myocytes, play an important role in muscle wasting [28,29]. Therefore, we investigated the effect of ajoene extract on the level of myokines such as IL-6 and myostatin in muscles of tumor-bearing mice. IL-6 mRNA expression showed the tendency to increase in the tumor control group and to decrease by ajoene extract treatment (Figure 2A). We also determined the level of interleukin-6 receptor (IL-6R) since it is involved in IL-6 stabilization and IL-6 signaling activation [30]. Similarly, mRNA expressions of IL-6R and myostatin were increased in the tumor control group, whereas they were significantly reduced in response to ajoene extract treatment (IL-6R, *p* = 0.042; myostatin, *p* < 0.001) (Figure 2A,B). Given that IL-6 is released by myeloid cells including macrophages and myeloid-derived suppressor cells (MDSCs) associated with cachexia [31,32,33], we analyzed the proportions of macrophages and MDSCs in the spleen. The proportions of both were elevated in the tumor control group compared with the control group (*p* < 0.01), while they were significantly suppressed in the 10 mg/kg ajoene extract-treated mice (macrophages, *p* < 0.05; MDSCs, *p* < 0.01) (Figure 2C). These results were in agreement with the reduced mRNA levels of IL-6 and IL-6R in respective groups (Figure 2A). Taken together, our data suggest that ajoene extract effectively suppresses myokine secretion in muscles of tumor-bearing mice, thereby contributing to the protection against cancer-induced muscle wasting. 

### 3.3. Ajoene Extract Inhibits Muscle Degradation by Down-Regulating JAK/STAT3 and SMADs/FoxO Signaling Pathways In CT26 Tumor-Bearing Mice

**Figure 3:**
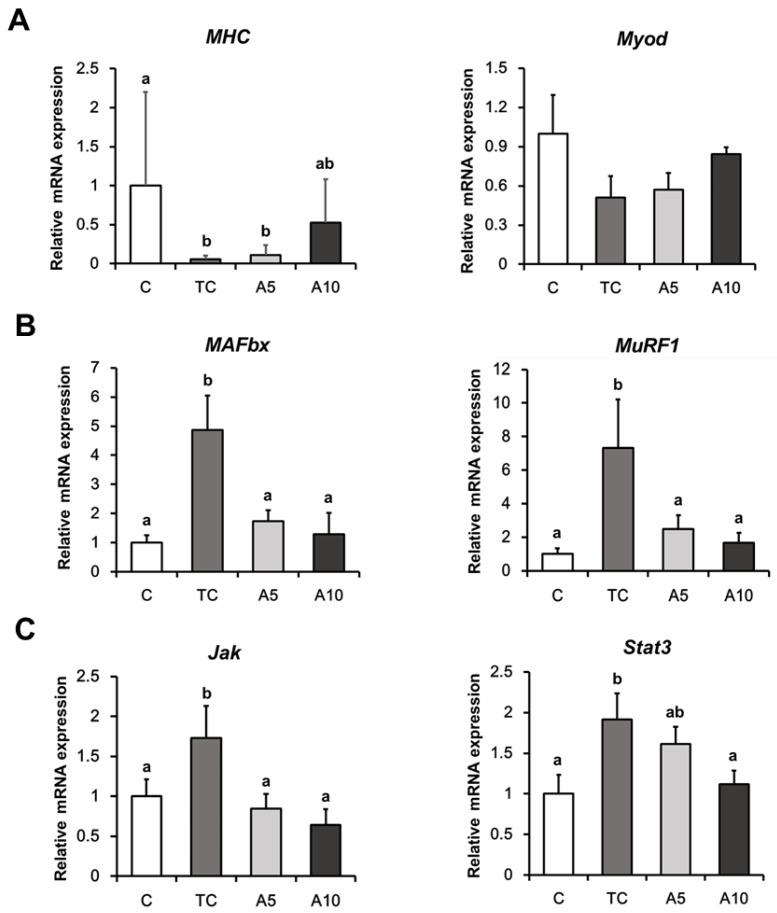

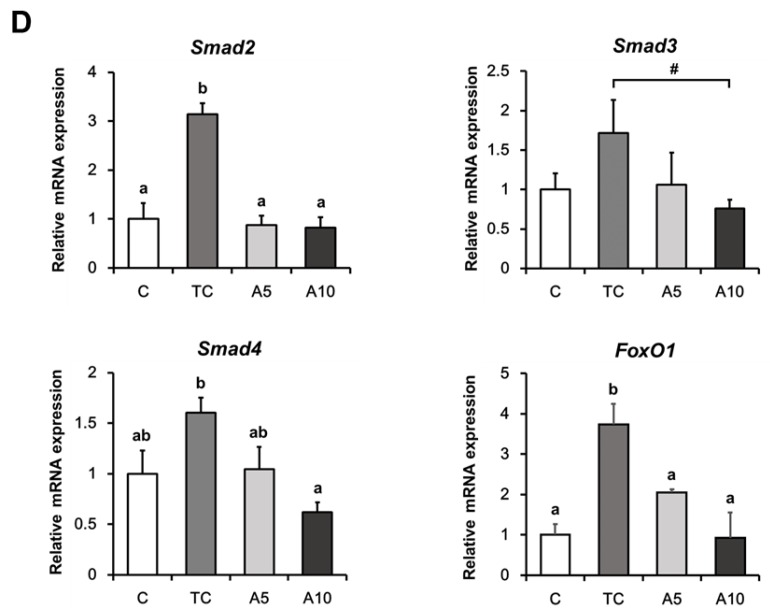
Effects of ajoene extract on muscle atrophy-associated markers in CT26 tumor-bearing mice. The mRNA expression levels of genes associated with myotube synthesis (**A**), muscle degradation (**B**), JAK/STAT3 (**C**), and SMADs/FoxO signaling pathways (**D**). Values are presented as the mean ± SEM. Statistical significance was evaluated by one-way ANOVA followed by the Duncan’s multiple range test or Student’s t-test. Means with different superscript letters are significantly different at *p* < 0.05 (**A**, **C**, **D**) and *p* < 0.01 (**B**). # *p* < 0.05 compared with the TC group. C, control; TC, tumor control; A5, 5 mg/kg Z-ajoene; A10, 10 mg/kg Z-ajoene (*n* = 5 per group).

To investigate the effects of ajoene extract on muscle wasting in tumor-bearing mice, we determined the mRNA expression levels of genes associated with myotube synthesis and muscle degradation. Myosin heavy chain (MHC) expression was significantly decreased in the tumor group, while it tended to increase in the 10 mg/kg ajoene extract group (*p* = 0.039). The expression of MyoD, a myogenesis initiator, exhibited a similar pattern to MHC expression; however, the difference was not statistically significant (Figure 3A). E3 ligases, MAFbx and MuRF1 associated with the development of muscle catabolism [34] were elevated in the tumor control group, whereas ajoene extract treatment significantly decreased the expressions of these markers (MAFbx, *p* = 0.004; MuRF1, *p* = 0.003) (Figure 3B). 

Mice treated with ajoene extract exhibited more obvious changes in muscle degradation than myotube formation. Therefore, we further investigated molecular mechanisms underlying the protective role of ajoene extract in attenuating muscle degradation. IL-6 is known to activate JAK/STAT3 signaling to induce muscle-specific E3 ligases [4,10]. We observed elevated levels of Jak and Stat3 mRNA in the tumor control group, which were significantly reduced in the 10 mg/kg ajoene extract-treated mice (Jak, *p* = 0.029; Stat3, *p* = 0.046) (Figure 3C). 

Furthermore, myostatin activates the assembly of SMAD2/3 and SMAD4, which both, in turn, relocate into nucleus to stimulate transcription of muscle atrophy-related genes [35,36]. Therefore, we also analyzed myostatin-related signaling as another upstream pathway of MAFbx and MuRF1. As with the genes involved in the JAK/STAT3 pathway, 10 mg/kg ajoene extract significantly suppressed the expression of Smad2, Smad3, and Smad4 compared with the tumor control group (Smad2, *p* = 0.012; Smad3, *p* = 0.049; Smad4, *p* < 0.001) (Figure 3D). Ajoene extract also significantly down-regulated the expression of FoxO1, which is activated by Smad2 and Smad3 (*p* < 0.001) (Figure 3D). Collectively, these results indicate that ajoene extract alleviates muscle atrophy by modulating JAK/STAT3 and SMADs/FoxO signaling pathways in tumor-bearing mice.

### 3.4. Z-ajoene Stimulates Myogenesis

**Figure 4:**
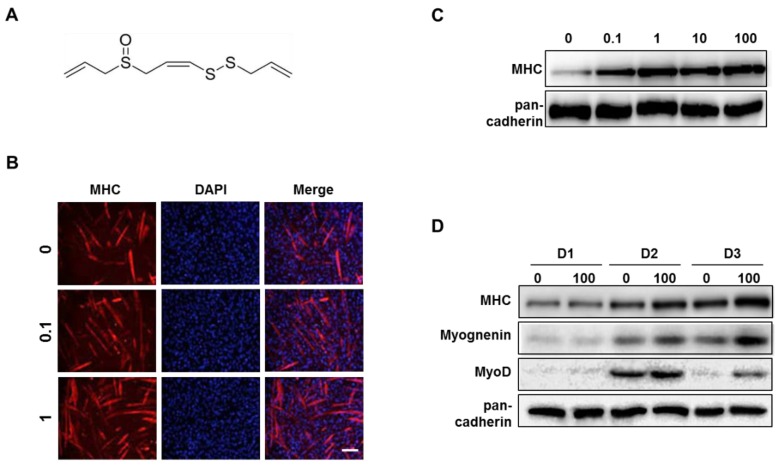
Effect of Z-ajoene on myoblast differentiation. (**A**) Structure of Z-ajoene. (**B**) C2C12 myoblasts were differentiated using differentiation medium (DM) supplementary with Z-ajoene (0.1, 1, 10, or 100 nM) for 3 days and then collected for immunostaining of myosin heavy chain (MHC) (red) and 4’,6-diamidino-2-phenylindole (DAPI, blue). Scale bar = 200 μm. (**C**) Differentiated C2C12 cells in the presence of Z-ajoene were subjected to Western blot analysis to determine the expression level of MHC. (**D**) Day-dependent effect of Z-ajoene (100 nM) on expression levels of myogenic factors during myoblast differentiation.

Normally, during differentiation after addition of differentiation medium (DM), mononucleated myoblasts become long and tubular myocytes. These myocytes fuse together to become multi-nucleated and adopted the cylinder-shape of myotubes [37], and mature myotubes can be detected by immunostaining for MHC and 4’-6-diamidino-2-phenylindole (DAPI). We observed increased myoD expression and a large number of mature myotubes after treatment of ajoene extract (Figure S3) indicating the myogenic effects. To investigate whether Z-ajoene (Figure 4A), as an active ingredient of garlic, has myogenic properties, we added it to myoblasts during differentiation. Z-ajoene increased MHC expression and formation of multinucleated mature myotubes in a dose-dependent manner (Figure 4B,C). Z-ajoene (100 nM) also day-dependently increased the expressions of MHC and myogenin as compared with respective day-specific control. The expression level of MyoD, a myogenic transcriptional factor, reached a maximum on differentiation day 2 (D2) in both control and Z-ajoene-treated cells. Exposure to Z-ajoene led to a 1.5-fold increase in the expression of MyoD as compared with control at D2 (Figure 4D). Taken together, Z-ajoene stimulates myoblast differentiation. 

### 3.5. Z-ajoene Activates p38 MAPK During Myogenesis

**Figure 5:**
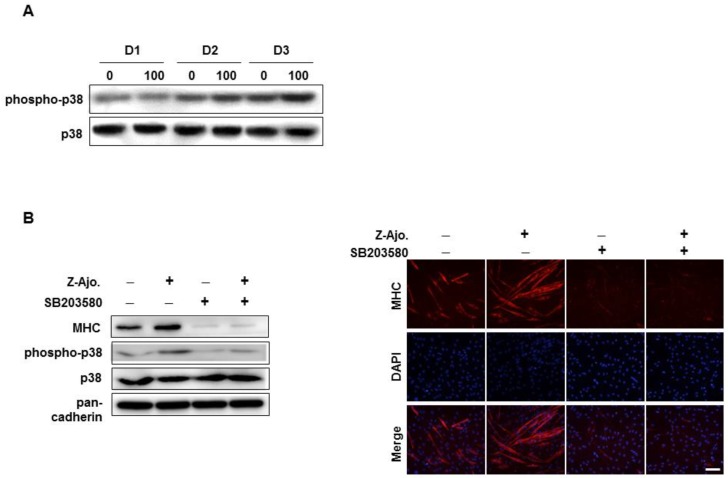
Effect of Z-ajoene on p38 MAPK activation during myoblast differentiation. (**A**) C2C12 cells were treated with Z-ajoene (100 nM) during days 1–3 of differentiation (D1–D3). Collected cell lysates were subjected to immunoblot. (**B**) C2C12 cells were pre-treated with (+) or without (-) SB203580 (10 μM) prior to Z-ajoene and differentiated in DM for 2 days. Cell lysates were subjected to Western blot analysis and immunostained with MHC (red) and DAPI (blue). Scale bar = 200 μm.

As the p38 mitogen-activated protein kinase (MAPK) activation is the most well-known mechanism in myoblast differentiation [38], we estimated the level of phosphorylated p38 MAPK during myogenesis. Phosphorylated-p38 MAPK continuously increased during the differentiation period and reached its highest level at D3. Z-ajoene treatment significantly activated p38 MAPK compared with control, demonstrating that its role in myoblast differentiation operates via activation of p38 MAPK (Figure 5A). 

Pre-treatment of SB203580 (10 μM, an inhibitor of p38 MAPK) prior to Z-ajoene treatment inhibited p38 MAPK phosphorylation by 43% (Figure 5A), and dramatically suppressed MHC expression and myotube formation (Figure 5B) compared with Z-ajoene group. These results suggest that p38 MAPK activation contributes to the Z-ajoene-induced myogenesis. 

### 3.6. Z-ajoene Prevents Myotube Protein Loss in Vitro

To investigate the preventive potential of Z-ajoene for muscle wasting, fully differentiated myotubes were treated with Z-ajoene (0.1 and 1 μM) prior to further treatment with conditioned medium (CM) from CT26 murine colon cancer cells [39]. The CM of cancer cells is known to create an inflammatory condition, associated with the production of pro-inflammatory cytokines in vitro and in vivo. These environments can trigger NF-κB activation following E3 ligases (MAFbx, MuRF1) expression to cause muscle atrophy [39]. 

**Figure 6:**
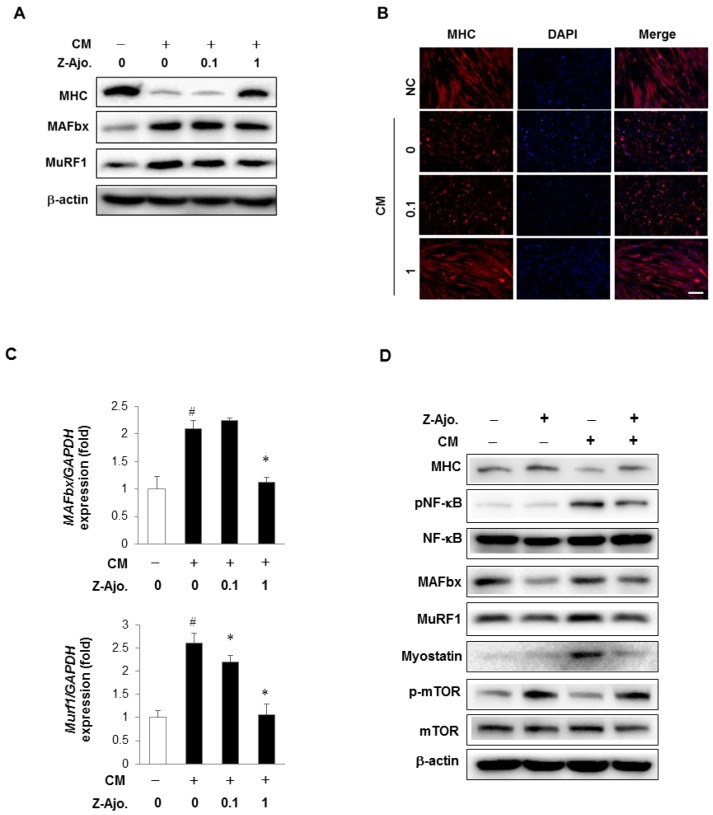
Preventive effect of Z-ajoene in conditioned media (CM) from CT26 colon carcinoma-induced myotube loss. C2C12 myoblasts were differentiated into myotubes for 3 days using differentiation medium (DM). Differentiated myotubes were pre-treated with Z-ajoene (1 μM) for 3 h, and then treated with (+) or without (-) 30% CM for 24 h. Cells were collected and the muscle specific E3 ligases expression was assessed by Western blot analysis (**A**) and quantitative real-time polymerase chain reaction (PCR) (**C**). Data are means ± SD of triplicated experiments. # *p* < 0.0001 compared with group treated only with DM; * *p* < 0.0001 compared with cells treated with CM. (**B**) Collected cells were fixed and immunostained with MHC (red) and DAPI (blue). Scale bar = 200 μm. (**D**) Cell lysates were subjected to Western blot analysis to analyze MHC, phosphorylated nuclear transcription factor kappa B (NF-κB), E3 ligases, myostatin, and phosphorylated mammalian target of rapamycin (mTOR).

CM decreased MHC level in differentiated myotubes, but 1 μM Z-ajoene recovered MHC levels by 4.3-fold compared with CM alone (Figure 6A). CM treatment increased the protein and mRNA levels of E3 ligases, MAFbx and MuRF1 in myotubes. However, pre-treatment of 1 μM Z-ajoene significantly reduced protein and mRNA expression of MAFbx and MuRF1 (Figure 6A,C). However, 0.1 μM Z-ajoene treatment could not suppress the expression of E3 ligases (Figure 6A,C). As shown in Figure 6B, Z-ajoene protected the loss of MHC expressing and multinucleated myotubes by attenuating the expression of MAFbx and MuRF1. Greater than 10 times higher concentration of Z-ajoene was needed for anti-myopathy activity when compared with concentration that elicited myogenic activity. The difference in effective concentrations of Z-ajoene may derive from the difference between normal differentiation condition and a damaged myotube environment.

As the balance between the rate of protein synthesis and protein degradation is important in the maintenance of skeletal muscle mass, we observed the effect of Z-ajoene on the expression of several factors that have been considered to mediate catabolism or anabolism of muscle proteins. Cancer mediated myotube atrophy is known to be induced by several catabolic mediators, such as E3 ubiquitin ligases, NF-κB, and myostatin [40]. We found that these mediators were increased by CM treatment, but significantly diminished in Z-ajoene-treated myotubes (Figure 6D). The mammalian target of rapamycin (mTOR) signaling pathway has been suggested to be an important anabolic pathway in muscle to increase skeletal muscle mass and fiber size [7]. Pre-treatment of Z-ajoene restored the decreased level of phosphorylated mTOR by CM damage. 

These data suggested that pre-treatment with Z-ajoene effectively prevented the CM-induced myotube loss, via regulation of both catabolic and anabolic pathways.

## 4. Discussion

Muscle atrophy is defined as reduced muscle fiber cross-sectional area, protein content, muscle strength, and insulin sensitivity [4,6,41]. Currently, the incidence of muscle atrophy is expected to increase due to increases in the elderly population (sarcopenia) and prevalence of chronic diseases and sedentary lifestyles (cachexia) [42]. About 20–40% of cancer patients die from muscle loss generated by cancer, not from the cancer itself [3,5]. Cachexia is caused by cancer, diabetes, obstructive pulmonary disease, acquired immune deficiency syndrome (AIDS), and chronic kidney failure. The main symptoms of cachexia are skeletal muscle wasting, anorexia, and unintentional weight loss, leading to progressive functional impairments [2,3,4]. To date, no effective treatment for the pharmacological management of cachexia exists due to multiple underlying biological mechanisms [43]. Sarcopenia is an age-related muscle wasting condition which was recently recognized as a new clinical disease by International Classification of Disease in 2016. To overcome muscle atrophy, we need to discover agents that can protect muscle against cachexic stress and/or enhance the differentiation of myoblasts into myotubes. 

CT26 colorectal adenocarcinoma bearing mice are commonly used to induce cancer cachexia with several accompanying symptoms including hepatic functional impairments, adipose and skeletal muscle wasting, and an increase in IL-6 concentration [44,45]. Our in vivo results showed that ajoene extract (14.2% *w*/*w* ajoene, 10 mg/kg body weight) effectively decreased muscle atrophy by reducing secretion of myokines such as IL-6 and myostatin and down-regulating JAK/STAT3 and SMADs/FoxO signaling pathways, thereby suppressing muscle-specific E3 ligases. These results were observed at the dose that did not exhibit anti-cancer effects in CT26 tumor-bearing mice, indicating muscle-specific activity of ajoene extract.

Ajoene extract was prepared from garlic bulb through crushing, heat treatment and organic solvent extraction. We analyzed composition of the extract and identified Z-ajoene as the main constituent (11.1% *w*/*w*) that might also contain linear polysulfides and vinyldithiin [46]. We previously reported several biological activities of Z-ajoene, including activation of Nrf2 and suppression of NK-kB signaling that could contribute to anti-myopathy potential of Z-ajoene. [22,24]. In order to confirm the preventive activity and uncover the mechanisms behind the effects of ajoene extract on muscle atrophy, we used pure Z-ajoene as the main component of the extract. Conditioned medium (CM) from cancer cell culture has been well-established as an inducer of cachexic conditions. We treated mature myotubes with CM derived from CT26 colon carcinoma cells to mimic in vitro cachexia. Z-ajoene protected against cachexic damage by modulating catabolism or anabolism of muscle proteins. Z-ajoene protected against muscle degradation through suppressing the levels of E3 ubiquitin ligases (MAFbx, MuRF1) [47], NF-κB, and myostatin [40]. Under cachexic conditions, myo-proteins become substrates for NF-κB-mediated E3 ubiquitin ligases resulting in proteasomal degradation. Although many studies report that inhibitors of proteasomal degradation can attenuate myo-protein degradation, there are no clinical applications of these inhibitors for treatment of muscle wasting diseases [34,48,49]. Myostatin, a TGFβ family member, functions in an autocrine-manner to balance muscle growth under normal conditions [50,51]. As excessive levels of myostatin mediate muscle atrophy [35], myostatin inhibition may prevent muscle loss in cachexic conditions. Many studies have demonstrated that NF-κB upregulates myostatin gene expression via direct binding to promoter regions in cachexic conditions [52,53].

Additionally, Z-ajoene positively regulated an anabolic pathway of protein synthesis. Z-ajoene restored the decreased level of phosphorylated mTOR by CM-induced decreases in mature myotubes. Taken together, the results from in vivo and in vitro studies indicate that ajoene extract and Z-ajoene can prevent cancer-induced muscle atrophy by suppressing cellular pathways associated with muscle protein degradation.

As a strategy for muscle regeneration in atrophic conditions, we evaluated the effect of Z-ajoene on myoblast differentiation into myotubes. Activated myogenic satellite cells undergo myogenesis to become myoblasts, and then fuse together to form myotubes. The myotubes then cluster into myofibers to form muscle. Considering the role of satellite cells on muscle generation, myogenesis-stimulating compounds may be able to function as potential therapeutic agents for treating muscle atrophy. C2C12 cells are mouse skeletal myoblasts derived from muscle satellite cells, and can be differentiated by serum starvation medium. The myoD and myogenic regulatory factor (Myf)-5 not only contribute to myogenic lineage specification of muscle stem cells, but also induce the expression of myogenin and myogenic regulatory factors (MRFs), leading to terminal differentiation [54,55]. In particular, the interaction of MyoD as a myogenic transcriptional factor with non-muscle specific muscle proteins is essential to the expression of myogenin and MHC, following new myofiber formation. Z-ajoene enhanced the expression of myogenic factors including MyoD, MHC or myogenin both in vivo and in vitro and increased the formation of multinucleated myotubes during myoblast differentiation. To clarify the mechanism of Z-ajoene-mediated myogenesis, we investigated the p38 mitogen-activated protein kinase (MAPK) that is considered as one of the key regulators of myoblast differentiation. It has been shown that binding of phosphorylated E proteins, SWI/SNF subunit BAF60, or Mef2 by p38 MAPK to myoD contributes to expression of myogenic factors [56]. Consistent with other studies, p38 MAPK phosphorylation gradually increased with differentiation day and was potentiated by Z-ajoene treatment, while SB203580, a p38MAPK inhibitor counteracted the Z-ajoene-induced myogenesis.

## 5. Conclusions

**Figure 7:**
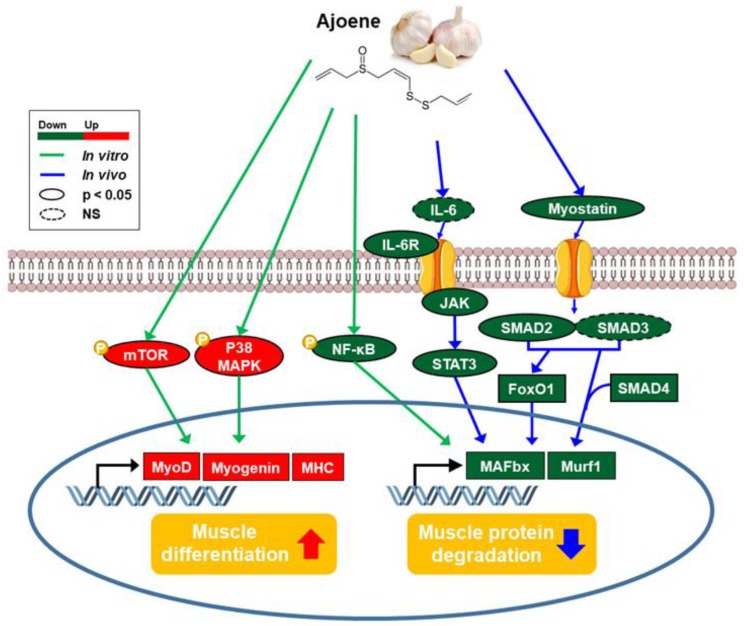
Summary of the effects of Z-ajoene from *Allium sativum* on muscle atrophy and related molecular mechanisms in vivo and in vitro.

In the present study, ajoene extract from crushed garlic (*Allium sativum*) ameliorates muscle atrophy by down-regulating not only myokines secretion but JAK/STAT3 and SMADs/FoxO signaling pathways, contributing to the preservation of muscle mass in a mouse model of cancer-induced cachexia. Z-ajoene not only attenuates myo-protein degradation under cancer-induced muscle wasting, but also stimulates myogenesis (Figure 7). Therefore, we suggest that Z-ajoene extracted from garlic has potential as a nutritional supplement for the prevention and treatment of muscle atrophy for cancer patients. 

## Figures and Tables

**Table 1:** Tissues weights at the harvest in CT26 tumor-bearing mice.

	C	TC	A5	A10	*p*-Value
Gastrocnemius (g)	0.1297 ± 0.0048	0.1244 ± 0.0029	0.1278 ± 0.0038	0.1324 ± 0.0031	0.543
Tibialis (g)	0.0256 ± 0.0038	0.0192 ± 0.0030	0.0237 ± 0.0026	0.0236 ± 0.0018	0.509
Extensor digitorum longus (g)	0.0161 ± 0.0008	0.0143 ± 0.0005	0.0167 ± 0.0008	0.0144 ± 0.0007	0.058
Soleus (g)	0.0082 ± 0.0008	0.0088 ± 0.0004	0.0080 ± 0.0008	0.0092 ± 0.0009	0.662
Quadriceps (g)	0.1365 ± 0.0068	0.1172 ± 0.0050	0.1349 ± 0.0062	0.1402 ± 0.0080	0.109
Extra muscle (g)	0.2269 ± 0.0105	0.1902 ± 0.0112	0.2175 ± 0.0142	0.2350 ± 0.0142	0.101
Total muscle (g)	0.5592 ± 0.0183 ^a^	0.4804 ± 0.0131 ^b^	0.5254 ± 0.0158 ^ab^	0.5562 ± 0.0196 ^a^	0.011
Heart (g)	0.13 ± 0.00	0.13 ± 0.01	0.12 ± 0.00	0.12 ± 0.00	0.394
Spleen (g)	0.12 ± 0.01 ^a^	0.28 ± 0.02 ^b^	0.27 ± 0.02 ^b^	0.27 ± 0.02 ^b^	<0.0001
Liver (g)	1.10 ± 0.06 ^a^	1.35 ± 0.05 ^b^	1.41 ± 0.05 ^b^	1.48 ± 0.04 ^b^	<0.0001
Epididymal fat (g)	0.34 ± 0.02	0.25 ± 0.03	0.27 ± 0.03	0.30 ± 0.03	0.127
Mesenteric fat (g)	0.21 ± 0.02	0.19 ± 0.02	0.18 ± 0.02	0.17 ± 0.02	0.452
Perirenal fat (g)	0.09 ± 0.01 ^a^	0.05 ± 0.00 ^b^	0.07 ± 0.01 ^ab^	0.06 ± 0.01 ^ab^	0.020
Total fat (g)	0.65 ± 0.04	0.46 ± 0.05	0.52 ± 0.06	0.55 ± 0.06	0.193

Data are presented as mean ± SEM. Values with different letters significantly differ at *p* < 0.05. C, control; TC, tumor control; A5, ajoene 5 mg/kg; A10, ajoene 10 mg/kg (*n* = 10 per group).

## Supplementary Materials

The following are available online at https://www.mdpi.com/2072-6643/11/11/2724/s1, Figure S1: Chromatogram of ajoene extract in high performance liquid chromatography (HPLC), Figure S2: Experimental design of mouse model of cancer cachexia, Figure S3: Effect of ajoene extract on myoblast differentiation, Table S1: Oligonucleotide primer sequences used for the real-time quantitative polymerase chain reaction (qRT-PCR) analysis.
