# Supplementary material for: Z-ajoene from Crushed Garlic Alleviates Cancer-Induced Skeletal Muscle Atrophy
Source: Nutrients. 2019 Nov 10;11(11):2724. doi: 10.3390/nu11112724 (PMC6893518; doi:10.3390/nu11112724)
Supplement: Supplementary file 1 [file nutrients-11-02724-s001.pdf]

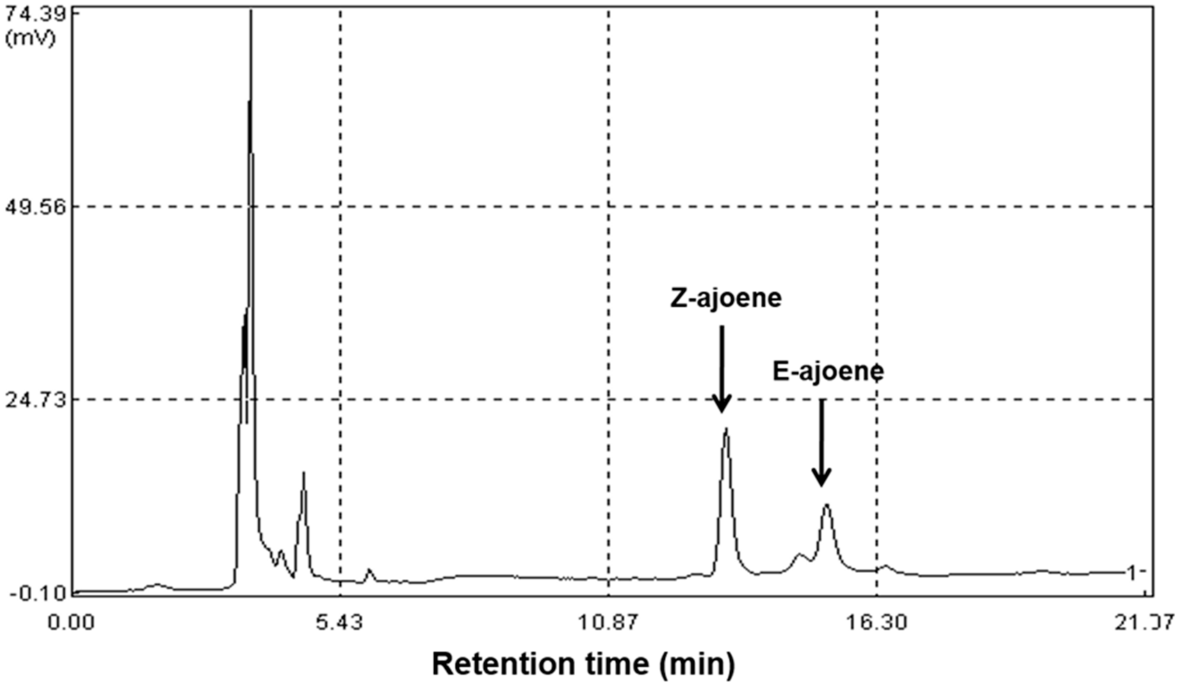

Figure 1. Chromatogram of ajoene extract in high performance liquid chromatography (HPLC). HPLC conditions are as follows, column: Spherisorb silica, 5 μm, 4.6×250 mm, eluent: n-hexane/10% iso-propyl alcohol in ethyl acetate (67/33) 1 ml/min, detection 254 nm UV.

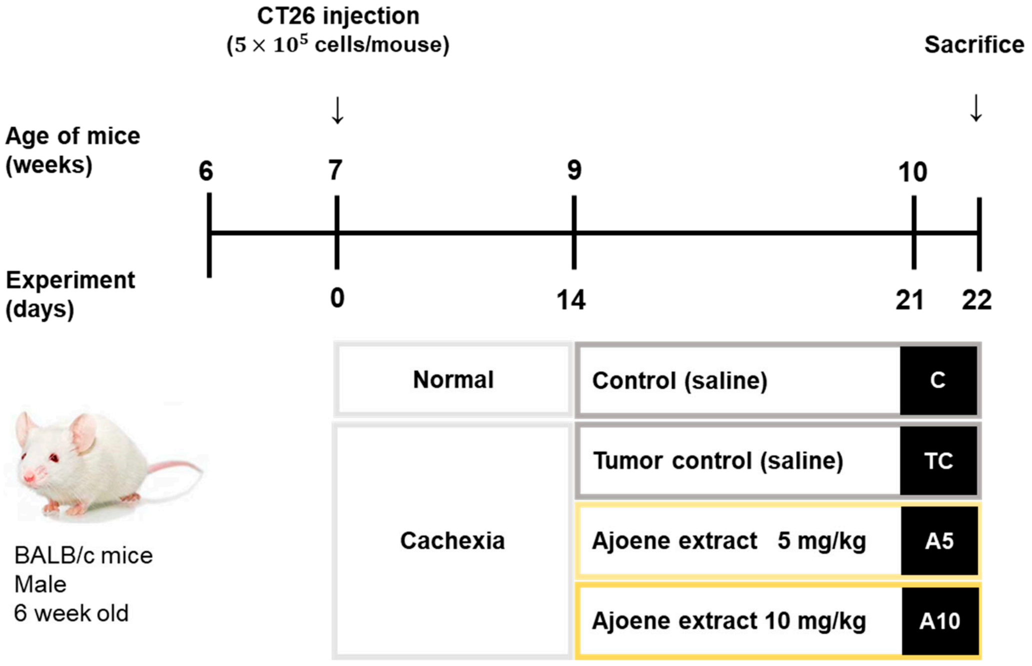

Figure 2. Experimental design of mouse model of cancer cachexia.

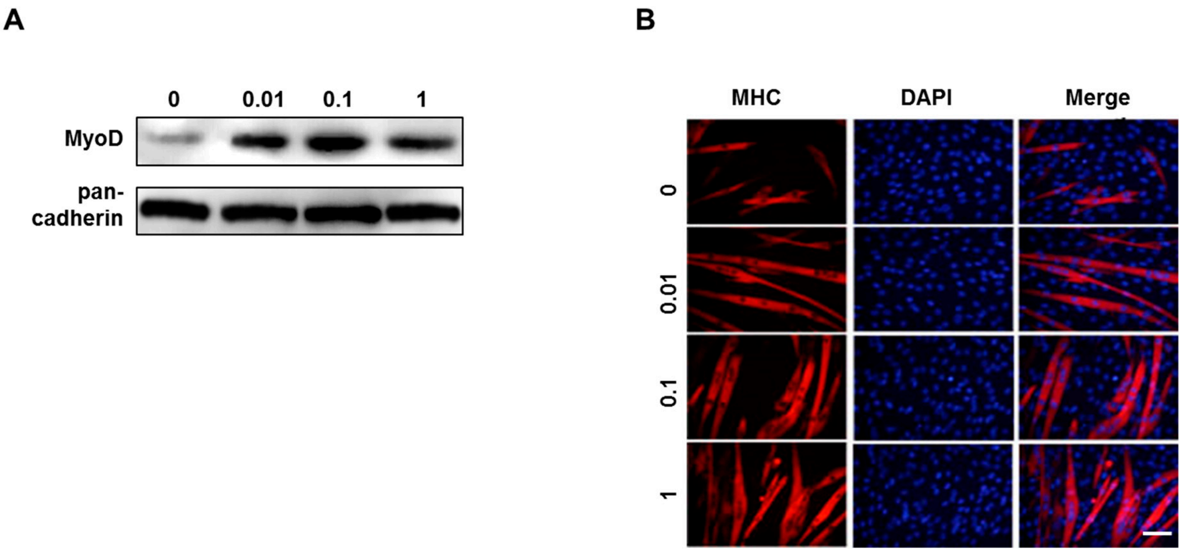

Figure 3. Effect of ajoene extract on myoblast differentiation. C2C12 cells were differentiated using differentiation medium (DM) supplemented with ajoene extract (0.01, 0.1, or 1 μg/mL) for 3 days, and then cells were collected to perform Western blotting analysis to determine the level of myoD expression (A) and immunostaining of MHC (red) and 4',6-diamidino-2-phenylindole (DAPI, blue) (B). Scale bar = 200 μm.

Table 1. Oligonucleotide primer sequences used for the real-time quantitative polymerase chain reaction (qRT-PCR) analysis.

Gene	Forward Primer	Reverse Primer	Accession Number
In vitro			
MAFbx	CGACCTGCCTGTGTGCTTAC	CTTGCGAATCTGCCTCTCTG	BC027211
MuRF1	GGTGCCTACTTGCTCCTTGT	CTGGTGGCTATTCTCCTTGG	NC_000070
GAPDH	TGCACCACCAACTGCTTAG	GGCATGGACTGTGGTCATGAG	BC096042
In vivo			
FoxO1	CAAAGTACACATACGGCCAATCC	CGTAACTTGATTTGCTGTCCTGAA	NM_019739.3
IL-6	TTTCCTCTGGTCTTCTGGAGT	GTGACTCCAGCTTATCTCTTG	NM_001311144.1
IL-6R	ATGATGCCTTGCGAGGAGTG	TGGGCTCTATCCAAGGAGTG	NM_001310676.1
Jak	GGAGTACTACACAGTCAAGGACGA	AAACATTCCGGAGCGTACC	NM_013567.1
MAFbx	AGTGAGGACCGGCTACTGTG	GATCAAACGCTTGCGAATCT	NM_026346.3
Mstn	CAGACCCGTCAAGACTCCTACA	CAGTGCCTGGGCTCATGTCAAG	NM_010834.3
MuRF1	TGACATCTACAAGCAGGAGTGC	TCGTCTTCGTGTTCCTTGC	NM_001369245.1
Myh	CTCAGGCTTCAAGATTTGGTGG	TTGTGCCTCTCTTCGGTCATTC	NM_001039545.2
MyoD	AAACCCCAATGCGATTTATCAGG	TAAGCTTCATCTTTTGGGCGTGA	NM_010866
Smad2	AAGCCATCACCACTCAGAATTG	CACTGATCTACCGTATTTGCTGT	XM_006525699.2
Smad3	GGGGAGCAGAGTACAGGAGA	CTGCAAGGGTCCATTCAGGT	NM_016769.4
Smad4	AAGGTGGGGAAAGTGAAAC	ATGCTTTAGTTCATTCTTGTG	NM_001364968.1
Stat3	TTTCATCAGCAAGGAGCGGG	GGGGTAGAGGTAGACAAGTGGA	NM_011486.5
GAPDH	CAACTTTGGCATTGTGGAAG	CTGCTTCACCACCTTCTTG	NM_001289726.1

FoxO1, forkhead box protein O1; *IL-6*, interleukin 6; *IL-6R*, interleukin 6 receptor; *Jak*, janus kinase; *MAFbx*, muscle atrophy F-box (MAFbx/atrogin-1); *Mstn*, myostatin; *MuRF1*, muscle-specific RING finger protein-1; *Myh*, myosin heavy chain; *MyoD*, myoblast determination protein 1; *Smad2*, SMAD family member 2; *Smad3*, SMAD family member 3; *Smad4*, SMAD family member 4; *GAPDH*, glyceraldehyde 3-phosphate dehydrogenase.
